# An efficient cyber-attack detection and classification in IoT networks with high-dimensional feature set using Levenberg-Marquardt optimized feedforward neural network

**DOI:** 10.1371/journal.pone.0333899

**Published:** 2025-10-24

**Authors:** Quxi Kuang, Xianglin Kuang

**Affiliations:** 1 Computer Science and Engineering, University of New South Wales, Sydney, New South Wales, Australia; 2 School of Tourism and E-commerce, Baise University, Baise, China; Northwestern Polytechnical University School of Software and Microelectronics, CHINA

## Abstract

This paper examines the escalating challenge of detecting cyber-attacks within Internet of Things (IoT) networks, where conventional security measures often falter in addressing the speed and complexity of contemporary threats. In response to the necessity for more precise, efficient, and adaptive security solutions, we propose a deep learning-based approach that employs feedforward neural networks optimized through the Levenberg-Marquardt algorithm. Our findings indicate that this method markedly surpasses traditional machine learning and deep learning models, such as Support Vector Machines (SVM), Random Forest, and Artificial Neural Network (ANN), achieving an accuracy rate of 99.7%, precision of 99.93%, recall of 99.93%, and an F1-score of 99.93%. Furthermore, the model demonstrates minimal misclassifications and effectively processes substantial data volumes, rendering it highly suitable for the real-time detection of various cyber threats. This system substantially reduces false positive rates and enhances the classification accuracy of different attack types within IoT networks. This research contributes to the advancement of cybersecurity in IoT environments by providing a scalable and robust solution for identifying emerging cyber threats.

## 1 Introduction

As technology advances rapidly, the integration of the Internet of Things (IoT), smart devices, and cloud computing has fundamentally transformed the way we connect, communicate, and operate in nearly every aspect of our lives [1]. From smart homes to automated industrial systems, the digital realm has become deeply interwoven into our daily routines. However, these advancements also bring significant security concerns, as malicious actors exploit vulnerabilities within these interconnected systems to launch devastating cyber-attacks [2]. The increase in cyber-attacks underscores the urgent need for more robust and intelligent security mechanisms to protect against these evolving threats. Within this framework, we explore the growing challenge of detecting cyber-attacks, particularly in highly dynamic and distributed environments [3].

Cyber-attacks have grown increasingly sophisticated over the past few decades, evolving from basic intrusions to highly complex and coordinated assaults that exploit vulnerabilities in systems and networks. These attacks can vary from simple denial-of-service (DoS) incidents to intricate, multi-layered advanced persistent threats (APTs) [4]. As our reliance on technology intensifies, cyber-attacks are no longer limited to personal devices or small businesses; they have escalated into threats capable of disrupting national infrastructure, corporate ecosystems, and personal privacy on an unprecedented scale [5].

Notably, the Internet of Things (IoT) has emerged as a prime target, with billions of devices connected to the Internet, each serving as a potential entry point for malicious actors [6]. Although traditional cybersecurity measures such as firewalls, antivirus software, and intrusion detection systems (IDS) have played a critical role in mitigating cyber threats, they are increasingly inadequate to cope with the speed and complexity of modern attacks [7]. Consequently, there is a growing demand for advanced, intelligent detection systems capable of adapting to new attack methods, identifying unknown threats, and minimizing false positives in real-time scenarios [8]. Artificial intelligence techniques, particularly those centered on machine learning and deep learning, have emerged as powerful tools to tackle these challenges. They enable the analysis of vast datasets and assist in identifying patterns that may indicate potential cyber threats [9].

Despite ongoing advancements in cybersecurity technologies, the pace of cyber-attacks is outstripping the capabilities of traditional detection systems. Signature-based methods often fall short in identifying new or zero-day attacks, while anomaly detection systems tend to generate high rates of false positives or overlook critical threats [10]. As network traffic continues to grow in complexity and volume, conventional systems struggle to effectively monitor and pinpoint potential threats in real-time. This situation highlights an urgent need for more accurate, efficient, and adaptable systems that can detect both known and unknown cyber-attacks [11]. This paper aims to bridge this gap by investigating how deep learning algorithms, specifically feedforward neural networks optimized with the Levenberg-Marquardt algorithm, can enhance the detection and classification of cyber-attacks in IoT networks. A primary challenge addressed in this research is the high dimensionality and complexity of data generated by IoT devices, which can overwhelm traditional detection systems. Furthermore, the diversity of cyber-attacks in IoT networks, marked by their varied attack patterns, complicates the ability of established approaches to classify and detect all potential threat types. As IoT networks continue to expand, the demand for scalable and intelligent systems that can accurately identify attacks with minimal human intervention becomes increasingly critical.

The motivation for this research arises from the urgent need to enhance cybersecurity mechanisms in an era characterized by the Internet of Things (IoT) and digital transformation. As organizations and individuals increasingly embrace new technologies, the potential risks associated with cyber-attacks escalate dramatically. A successful cyber-attack can lead to significant financial losses, data breaches, and irreversible harm to an organization’s reputation. Furthermore, the deployment of more IoT devices in sensitive sectors such as healthcare, smart cities, and industrial control systems poses the risk of catastrophic consequences, threatening public safety and national security.

Traditional cyber-attack detection methods tend to be reactive rather than proactive, relying on known attack signatures and predefined patterns. This strategy proves increasingly ineffective against novel attack techniques that have yet to be identified. Machine learning, particularly deep learning, provides an opportunity to overcome these limitations by enabling the analysis of large datasets, the detection of unknown attacks, and adaptation to emerging threats. This research is vital, as it holds the potential to bolster the security of IoT networks and contribute to the development of more resilient digital infrastructures. By employing advanced optimization techniques like the Levenberg-Marquardt algorithm within neural networks, the aim is to improve detection accuracy, reduce false positives, and create a more efficient system for real-time attack classification.

### 1.1 Our contribution

This paper presents a deep learning approach to detecting cyber-attacks in IoT networks, utilizing feedforward neural networks (FNN) optimized through the Levenberg-Marquardt (LM) algorithm. Our key contributions are as follows:

- We enhance the training process by implementing the LM algorithm, which significantly improves convergence speed and accuracy compared to traditional methods, thereby facilitating efficient learning from high-dimensional IoT data.
- Our model undergoes rigorous evaluation against algorithms such as ANN, SVM and Random Forest, demonstrating superior performance in terms of accuracy, precision, and recall.
- We showcase that our optimized model can effectively manage large volumes of data, making it well-suited for real-time cyber-attack detection in IoT environments.
- Our system delivers detailed classifications across various attack types, providing valuable insights into the nature of detected threats.

The paper is organized as follows: Sect 2: Literature Review provides an overview of existing cyber-attack detection methods and discusses the application of machine learning techniques in cybersecurity, highlighting their strengths and limitations. Sect 3: Materials and Methodology outlines the dataset used in this study, the feature selection, and the neural network architecture. It also describes the optimization process using the Levenberg-Marquardt algorithm. Sect 4: Results and Discussion presents the experimental results, including performance metrics, comparison with benchmark models, and an analysis of the model’s effectiveness in detecting and classifying cyber-attacks. Finally, Sect 5: Conclusion highlights the main findings of the study, explores their implications, and proposes directions for future research to enhance cyber-attack detection systems.

## 2 Literature review

In the paper [12] the authors propose a deep-learning-based system for detecting and classifying cyber-attacks in IoT communication networks, leveraging Convolutional Neural Networks (CNN). The objective is to enhance attack detection by classifying IoT network traffic using both binary (normal vs. anomaly) and multiclass (normal, DoS, probe, R2L, U2R) classifiers. The methodology involves three key subsystems: feature engineering (data preprocessing and encoding), feature learning (CNN-based training), and traffic classification (final detection and classification). The system’s performance was assessed using the NSL-KDD dataset, yielding strong results with an accuracy of over 99.3% for binary classification and 98.2% for multiclass classification. Key performance metrics, such as precision, recall, F1-score, and false alarm rate, were used for evaluation. However, the proposed system does have some limitations, particularly regarding computational cost, as the deep learning model’s complexity requires high-performance hardware, such as GPUs and multi-core CPUs.

The research explores anomaly detection for intrusion detection systems (IDS) using a sequential approach on the CIDDS-001 dataset in the paper [13]. The authors aim to enhance attack detection by comparing two perspectives: single-flow, using individual flow features, and multi-flow, leveraging flow sequences. The methodology involves the evaluation of three machine learning models: Random Forest (RF), Multi-Layer Perceptron (MLP), and Long Short-Term Memory (LSTM). Performance is assessed using various metrics, including accuracy, F1-score, recall, precision, and the false positive rate (FPR). Results show that the multi-flow approach, particularly with the LSTM model, significantly improves detection, with LSTM achieving an F1-score of 91.66% for a sequence of 70 flows. However, while LSTM performs well for sequential data, the RF model struggles as sequence length increases. The paper highlights the benefits of sequential analysis for anomaly detection but also notes challenges in computational cost and data preparation for larger datasets.

In the paper [14], the authors investigate the application of deep learning models for detecting cyber-attacks in IoT networks, using the CICIoT2023 dataset. The primary goal was to evaluate the performance of various deep learning models, including RNN, CNN, and DNN, for multi-class classification and attack detection. Their methodology involved preprocessing the data with robust scaling and label encoding techniques before training the models on the processed data. Performance was assessed using metrics such as accuracy, recall, precision, and F1-score. The results revealed that the RNN model outperformed the other models, achieving the highest accuracy of 96.56%, along with superior precision and recall. However, the paper also highlights some challenges, including the high computational complexity of deep learning models, particularly in real-time applications.

The authors of the paper [15] compare ten supervised machine learning algorithms for detecting and classifying attacks in IoT networks, using the CICIoT2023 dataset, which includes data from 105 devices and 33 attack types. The study evaluates algorithms like Random Forest (RF), XGBoost, CNN, LSTM, and others based on performance metrics such as accuracy, F1-score, recall, and precision. The results show that RF and XGBoost lead with the highest precision and accuracy, while CNN and other deep learning models also show strong results but struggle with class imbalance. The paper highlights the importance of balancing precision and recall, as missed attacks in IoT can have severe consequences. However, the study also identifies limitations such as the impact of class imbalance on performance and the computational complexity of deep learning models, which might not be ideal for IoT environments with limited resources. The findings guide selecting the most appropriate algorithms for different IoT deployment scenarios.

In the study [16], the researchers presented a distributed deep learning (DL)-based attack detection framework for cyber-attacks in IoT networks, leveraging two models: Feed Forward Neural Network (FFNN) and Long Short-Term Memory (LSTM). The primary objective is to enhance IoT security by detecting various attack types through a distributed system that reduces latency and computational overhead. The framework utilizes two datasets, BoT-IoT and NSL-KDD, after performing preprocessing techniques such as feature selection and data balancing. Performance is evaluated using metrics like recall, precision, and accuracy. Results show that FFNN outperforms LSTM, particularly for IoT-specific attacks, with an accuracy of up to 99.97%. However, the distributed framework, while efficient, experiences slight performance degradation (1%–2%) as the number of fog nodes increases, highlighting challenges in managing distributed data variance.

In the paper [17], the researchers introduce a two-stage deep learning framework aimed at detecting and attributing cyber-attacks in Industrial Control Systems (ICS), with a particular focus on IoT-enabled cyber-physical systems. The first stage addresses attack detection by applying a novel ensemble deep representation learning model in combination with a decision tree (DT), which helps manage the challenges of imbalanced ICS datasets. In the second stage, attack attribution is improved using an ensemble of one-vs-all classifiers paired with a deep neural network (DNN) for classifying the attack’s characteristics. The model’s performance, assessed with metrics like precision, recall, accuracy, and F-measure, demonstrates its advantage over other methods, particularly in handling previously unseen attacks and refining attribution accuracy. However, the study acknowledges that despite these gains, the framework’s complexity remains similar to existing DNN-based models, with longer training and testing times as a result of the dataset size and the number of attack attributes.

The paper [18] presents a hybrid deep learning-based intrusion detection system (IDS) for the Industrial Internet of Things (IIoT), combining Convolutional Neural Networks (CNN) and Long Short-Term Memory (LSTM) models. The primary objective is to detect and classify cyber-attacks in IIoT environments using real traffic data from the Edge-IIoTset dataset. The model is evaluated using binary and multi-class classification methods, and performance is measured by accuracy, precision, false positive rate (FPR), and detection cost. The results show that the CNN-LSTM model outperforms other traditional machine learning models and standalone LSTM in terms of accuracy and precision, achieving 100% accuracy for binary classification. However, the hybrid deep learning model comes with higher detection costs compared to traditional methods. The limitations of the model include the computational cost associated with deep learning techniques, although it offers superior detection capabilities. This research highlights the effectiveness of combining CNN and LSTM for IIoT security and suggests future work to explore anomaly detection using various deep learning techniques.

The study in [19] evaluates five machine learning algorithms such as Logistic Regression (LR), Neural Network (NN), SVM, k-NN, and Decision Tree (DT) for detecting cyber anomalies in IoT networks using the Bot-IoT andToN-IoT datasets. The goal was to identify the most effective method for anomaly detection. Results showed that the Neural Network outperformed all other models, achieving the highest F1 score of 0.999, showing its high accuracy. Logistic Regression performed poorly with a low F1 score. Although k-NN, SVM, and Decision Tree models showed good results, they were slightly less effective than NN. Limitations included poor classification of certain attacks, like MITM, by some models. This research highlights the potential of Neural Networks for enhancing IoT security and suggests future work to refine these models and explore additional algorithms for broader applicability.

The paper [20] provides a comprehensive survey of deep learning (DL)-based techniques for detecting cyber-attacks in Cyber-Physical Systems (CPS). The research aims to enhance cybersecurity in CPS environments by applying DL models to identify different kinds of cyber threats. The authors introduce a six-step methodology for developing and evaluating DL-driven attack detection models, model customization, data acquisition, covering CPS scenario analysis, performance evaluation, cyber attack identification, and problem formulation. Performance metrics such as recall, precision, accuracy, false positive rate, and F1-score are used to assess model effectiveness. The survey highlights the potential of DL models like Long Short-Term Memory (LSTM), Autoencoders (AE), and Convolutional Neural Networks (CNN) in improving attack detection. Nevertheless, the study recognizes challenges such as high computational cost, the need for large datasets, and issues with real-time implementation in some DL models. Despite these limitations, the paper emphasizes the growing importance of DL in CPS cybersecurity and suggests future research directions to overcome these challenges.

In the paper [21], the authors explore filter-based feature selection techniques for cyber attack detection, a critical component in designing effective intrusion detection systems (IDS). Feature selection is essential in reducing computational costs and enhancing model performance by identifying the most relevant features from high-dimensional datasets. The filter-based approach, which evaluates feature relevance using statistical measures independent of classifiers, offers a lightweight and computationally efficient solution, particularly suited for resource-constrained environments like IoT. The surveys of existing studies, detailing key elements such as search algorithms and relevance measures. Search strategies like forward selection and backward elimination help identify optimal feature subsets, while relevance measures such as mutual information and Pearson correlation assess feature importance. The authors examine the performance of these techniques across several datasets, demonstrating their impact on detection accuracy and model efficiency. Despite their advantages, filter-based methods do not account for feature interactions, which can limit their performance in complex attack scenarios. Additionally, the reliance on specific relevance measures may introduce biases.

The literature reveals several efforts in leveraging deep learning and machine learning models for detecting cyber-attacks, especially in IoT environments. Many approaches use models like CNNs, LSTMs, and Random Forests to improve detection accuracy. However, these models often suffer from high computational costs and the challenge of handling complex, high-dimensional datasets efficiently. Many existing methods struggle with achieving real-time performance due to their dependency on powerful hardware and large-scale datasets. The need for a new scheme arises from the limitations of existing methods in effectively handling the complexity and high dimensionality of IoT network data. Traditional models, such as CNNs and LSTMs, while effective in many contexts, often suffer from high computational costs, slow convergence, and the requirement for powerful hardware to process vast amounts of network traffic. Additionally, these models struggle with providing real-time detection, which is crucial for IoT environments where immediate response to attacks is essential.

## 3 Materials and methodology

In this section, we provide a detailed description of the dataset used in this research, including its characteristics and preprocessing steps. In addition, we will present a comprehensive discussion of our proposed model, describing its architecture, methodologies, and key components.

### 3.1 Dataset description

The University of New South Wales in Australia has collected data on various cyber attacks. This dataset consists of network packets captured in the Cyber Range Lab at UNSW Canberra (https://research.unsw.edu.au/projects/unsw-nb15-dataset).

The dataset categorizes cyber attacks into nine types, each with distinct characteristics in the Table 1.

**Table 1:** Types of attacks and their descriptions.

Attack Type	Description
**Normal**	The “Normal” class represents benign or non-attack instances, distinguishing them from malicious or attack-related behaviors in the dataset.
**Fuzzers**	Injects random data into a system to evaluate its robustness and identify vulnerabilities.
**Analysis**	Analyzes a system to detect vulnerabilities and possible exploitation targets.
**Backdoors**	Creates a hidden entry point in a system for future unauthorized access.
**DoS (Denial of Service)**	Aims to disrupt a system’s normal operations, making it unavailable to users.
**Exploits**	Takes advantage of system vulnerabilities to gain unauthorized access.
**Generic**	Includes various attack types that don’t fit into the other specific categories.
**Reconnaissance**	Gathers information about a system to prepare for future attacks.
**Shellcode**	Execute harmful code, typically as shell scripts, on a system.
**Worms**	Malware that infects other systems and potentially causes harm.

In this study, we analyze network traffic features from a cybersecurity dataset where the label (1 = attack) identifies malicious activity. The correlation coefficients indicate how strongly each feature associates with attack instances. Below, we explain the key features in simple terms to clarify their roles in detecting cyber threats.

- **sttl (0.62)**: *Source Time-To-Live (TTL)*. A high TTL value suggests that packets have an unusually long lifespan, often seen in spoofed attacks or attempts to evade detection.
- **ct_state_ttl (0.48)**: *Connection State Tracking + TTL*. Monitors the duration and state of network connections (e.g., active, timed-out). Unusual patterns here may signal prolonged malicious activities like brute-force attacks.
- **state (0.46)**: *Connection State*. Flags specific states (e.g., half-open connections), which are common in SYN flood attacks.
- **ct_dst_sport_ltm (0.37)**: *Long-Term Traffic to Destination Port*. High values indicate repeated connections to a specific port, typical in port scanning or targeted exploits.
- **rate (0.34)**: *Packet/Connection Rate*. Sudden spikes in traffic rate often correlate with DDoS attacks.
- **swin (-0.36)**: *Source TCP Window Size*. Smaller window sizes may reflect manipulated traffic to avoid detection.
- **dload (-0.35)**: *Destination Traffic Load*. Lower volumes could indicate stealthy attacks (e.g., data exfiltration).
- **dwin (-0.34)**: *Destination TCP Window Size*. Similar to *swin*, reduced sizes may hint at malicious flows.
- **dmean (-0.3)**: *Average Packet Size to Destination*. Smaller packets (e.g., command-and-control signals) are often suspicious.
- **sinpkt (-0.16)**: *Source Inter-Packet Time*. Shorter intervals suggest automated attacks (e.g., botnets).
- **proto (0.008)**: *Protocol Type (e.g., TCP, UDP)*. Attacks span multiple protocols, reducing its predictive power.
- **is_ftp_login (-0.0088)**: *FTP Login Attempts*. Minimal correlation, though failed logins might still be contextually relevant.
- **tcprtt (-0.025)**: *TCP Round-Trip Time*. Slightly lower latency could indicate local-network attacks but is not a strong standalone indicator.
- **Source-side anomalies** (e.g., high TTL, abnormal connection states) are critical markers of attacks.
- **Destination metrics** (e.g., load, window size) often decrease during attacks, reflecting stealthy or low-volume malicious traffic.
- **Connection tracking features** (e.g., ct_*) help detect long-term attack patterns, such as port scanning or persistent threats.

**Fig 1:**
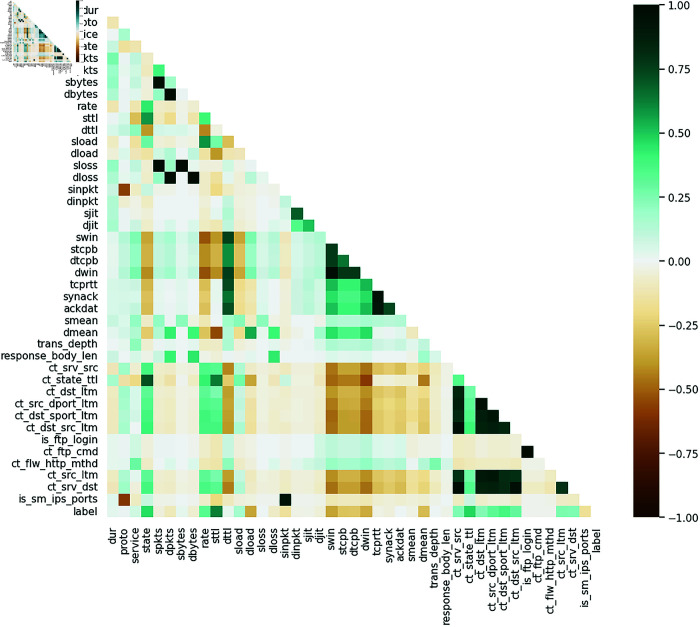
Correlation between variables and cyber attack (label).

This analysis underscores the importance of feature selection in enhancing cybersecurity frameworks. Fig 1 shows the analysis of the correlation between various variables and the occurrence of cyber attacks, considering the labeled data for identifying potential risk factors and attack patterns.

### 3.2 Network architecture

The neural network architecture proposed for classifying cyberattacks is a feedforward artificial neural network (ANN) with three layers: the input layer, the hidden layer, and the output layer. Each layer has a critical function in processing and classifying the input data. The design of the network is optimized to ensure effective performance in identifying various types of cyberattacks.

- **Input Layer:** The input layer of the network consists of 43 features, representing the input data used to classify the cyberattacks. These features are extracted from a dataset containing various characteristics of system logs, network traffic, or other relevant data. Each feature represents a distinct aspect of the system or network state, such as time duration, packet size, or type of protocol, and together they encapsulate critical information about the observed system behavior. The input layer’s neurons receive these values and pass them to the next layer, which processes the information further.
- **Hidden Layer:** The network’s hidden layer consists of 16 neurons, which are responsible for learning the complex relationships between the input features. These neurons utilize activation functions to transform the weighted sum of inputs from the previous layer into outputs. The choice of 16 neurons in the hidden layer was based on empirical experimentation, where this configuration provided an effective balance between network performance and complexity. A higher number of neurons in the hidden layer would result in increased computation time, while fewer neurons might lead to underfitting the data. The hidden layer plays a crucial role in extracting non-linear patterns and interactions within the data that are important for distinguishing between different cyberattacks.
- **Output Layer:** The output layer contains 10 neurons, each representing one of the 10 distinct classes of cyberattacks that the model is designed to identify. This layer takes the processed data from the hidden layer and produces a set of values that indicate the probability of each cyberattack class. These output values are then used for classification purposes, with the network assigning the most probable class to each input sample.

### 3.3 Optimization with Levenberg-Marquardt algorithm

We used *Levenberg-Marquardt (LM) algorithm* for optimizing our feedforward neural networks. It combines aspects of both *gradient descent* and the *Gauss-Newton method* to provide a more efficient and faster optimization technique. The LM algorithm adapts its behavior based on the landscape of the cost function and often converges faster than gradient descent. In this section, we provide a step-by-step breakdown of the optimization process using the Levenberg-Marquardt algorithm.

The first step is to define the error or cost function that we aim to minimize as given in the Eq 1.

$$J(𝐰)=\sum_{i=1}^{N}{\left({\hat{y}}_{i}-y_{i}\right)}^{2}$$ (1)

Where:

- J(𝐰) is the loss function,
- ${\hat{y}}_{i}$ is the predicted output of the network for input *x*_*i*_,
- *y*_*i*_ is the actual target value for input *x*_*i*_,
- *N* is the number of data points in the training set.

Next, the gradient of the loss function J(𝐰) with respect to the weights 𝐰 is computed. This involves calculating the first derivative of the error function. For a neural network, this is typically done using *backpropagation*, which propagates the error backward through the network to compute gradients as given in the Eq 2:

$$\nabla J(𝐰)=\left(\frac{\partial J(𝐰)}{\partial w_{1}},\frac{\partial J(𝐰)}{\partial w_{2}},\ldots ,\frac{\partial J(𝐰)}{\partial w_{m}}\right)$$ (2)

Where ∇J(𝐰) is the vector of gradients for each weight.

The next step is to compute the *Hessian matrix* 𝐇, which represents the second-order partial derivatives of the loss function with respect to the weights. In simpler terms, the Hessian matrix provides information about the curvature of the error surface. For a neural network, the Hessian is computed as given in the Eq 3:

$$𝐇=\sum_{i=1}^{N}\left(\frac{\partial {\hat{y}}_{i}}{\partial 𝐰}\right){\left(\frac{\partial {\hat{y}}_{i}}{\partial 𝐰}\right)}^{T}$$ (3)

Where:

- $\frac{\partial {\hat{y}}_{i}}{\partial 𝐰}$ is the Jacobian matrix of the predicted output ${\hat{y}}_{i}$ with respect to the weights 𝐰,
- The sum is taken over all data points.

The core idea behind the Levenberg-Marquardt algorithm is to modify the standard gradient descent update rule by incorporating a term from the Gauss-Newton method, which is effective for non-linear least-squares optimization. The update rule for the weight vector 𝐰 at iteration *k* is given in the Eq 4:

$${𝐰}_{k+1}={𝐰}_{k}-{\left(𝐇+\lambda 𝐈\right)}^{-1}\nabla J({𝐰}_{k})$$ (4)

Where:

- ${𝐰}_{k}$ is the weight vector at iteration *k*,
- 𝐇 is the Hessian matrix,
- λ is the *damping factor* that adjusts the contribution of the Gauss-Newton method and the gradient descent,
- 𝐈 is the identity matrix (this regularizes the Hessian to avoid singularities),
- $\nabla J({𝐰}_{k})$ is the gradient of the loss function at iteration *k*.

The damping factor λ plays a crucial role in balancing the *Gauss-Newton method* and *gradient descent*. Initially, a larger value of λ is chosen to take more conservative steps and prevent divergence. As the algorithm progresses, the damping factor is gradually reduced to allow for faster convergence. The process of updating the damping factor is as follows:

- When the algorithm takes a step and the error decreases, the damping factor λ is reduced to allow more aggressive steps.
- If the error increases, λ is increased to prevent divergence.

This adaptive adjustment of λ makes the Levenberg-Marquardt method highly effective for optimization in various landscapes.

The Levenberg-Marquardt algorithm iteratively updates the weights until the change in the loss function J(𝐰) is sufficiently small. A stopping criterion is defined in the Eq 5:

$$\left|J({𝐰}_{k+1})-J({𝐰}_{k})\right|<\epsilon$$ (5)

Where ϵ is a small tolerance value 10^−8^. Alternatively, the algorithm can stop when the maximum number of iterations is reached. The Algorithm 1 illustrates a step-by-step process of the proposed model.

**Algorithm 1** Levenberg-Marquardt Optimization for Neural Network Training

1: **Input:** Training data (X,Y), initial weights $w_{1},w_{2}$, damping

factor $\lambda_{0}$, max iterations max_iter, tolerance *ε*

2: **Output:** Optimized weights $w_{1}^{*},w_{2}^{*}$

3: Initialize weights and biases: *w*_1_, *w*_2_, *b*_1_, *b*_2_, $\lambda =\lambda_{0}$

4: **for** k = 1 to max_iter **do**

5:   **Forward Propagation:**

6:   Compute hidden layer: $H=f(W_{1}\cdot X+b_{1})$

7:   Compute output layer: $O=Softmax(W_{2}\cdot H+b_{2})$

8:   **Loss:** Compute loss $L=-\sum_{i=1}^{N}y_{i}\log(o_{i})$

9:   **Backpropagation:** Compute gradients:

10:   $Grad_{w_{2}}=H^{T}\cdot (O-Y)$

11:   $Grad_{w_{1}}=X^{T}\cdot ((O-Y)\cdot w_{2}^{T}\cdot f^{\prime }(H))$

12:   **Hessian:** $H=J^{T}\cdot J$

13:   Update weights:

14:   $\Delta w=-{\left(H+\lambda \cdot I\right)}^{-1}.\;Grad$

15:   $w_{1}=w_{1}+\Delta w_{1},w_{2}=w_{2}+\Delta w_{2}$

16:   **Update** *λ*:

17:   If loss decreases: λ=λ/10

18:   If loss increases: λ=λ·10

19:   **Check convergence:** If $|L_{k+1}-L_{k}|<\epsilon$, break

20: **end for**

21; **Return:** Optimized weights $w_{1}^{*},w_{2}^{*}$

## 4 Results and discussion

This section presents a comprehensive evaluation of the proposed model’s performance in detecting and classifying cyber-attacks in IoT networks. We provide an in-depth analysis of the model’s accuracy, precision, recall, and F1-score, comparing it against traditional machine learning algorithms

### 4.1 Error histogram

**Fig 2:**
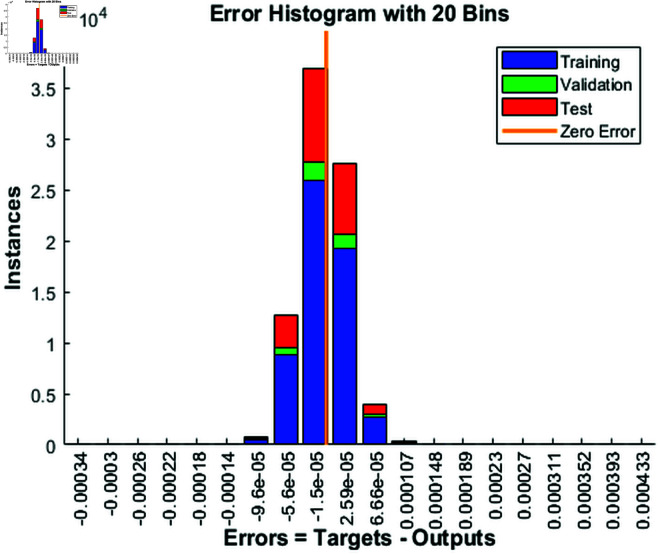
Error distribution of the proposed model for training, validation, and test datasets with zero error.

Fig 2 presents the distribution of errors from a proposed feedforward neural network model trained using the Levenberg-Marquardt algorithm. The errors, which are the differences between the target values and the model’s outputs, are plotted on the x-axis, while the y-axis shows the number of instances that fall within specific error ranges. The histogram is divided into four parts, each representing a different dataset: the blue bars correspond to the training data, the green bars represent the validation data, and the red bars show the test data. An orange vertical line marks the ideal zero error, where the model’s output perfectly matches the target values. The histogram indicates that most of the errors are close to zero, implying that the model performs well overall. The training, validation and test datasets have narrower error distributions, indicating that the model generalizes better to new, unseen data. While the majority of errors are small, this suggests that the Levenberg-Marquardt algorithm was effective in reducing error. Overall, the plot illustrates the neural network’s strong performance in predicting the target values, with minimal deviation from the ideal outcome.

### 4.2 Validation performance

**Fig 3:**
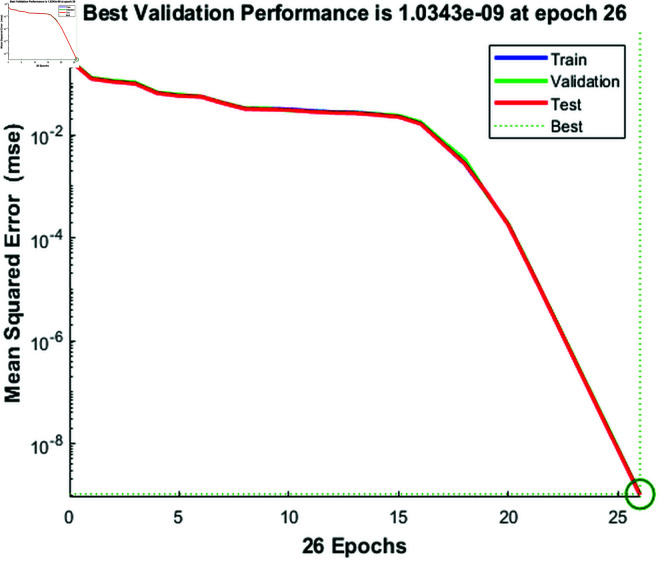
Training, validation, and test mean squared error across 26 epochs, with the best validation performance at epoch 26.

Fig 3 shows the performance of a feedforward neural network model trained using the Levenberg-Marquardt algorithm, with the mean squared error (MSE) plotted across 26 epochs for the training, validation, and test datasets. The x-axis represents the number of epochs, while the y-axis shows the mean squared error. The blue line represents the training dataset, the green line represents the validation dataset, and the red line represents the test dataset. The dotted green vertical line marks the epoch with the best validation performance, which occurs at epoch 26, with a validation MSE of 1.0343e-09. From the plot, we can observe that the training error (blue line) decreases rapidly at the beginning and continues to decline steadily throughout the training process. Both the validation (green line) and test (red line) errors also decrease, but the validation error stabilizes around epoch 25, while the test error remains relatively stable but slightly higher than the validation error. The marked best validation performance at epoch 26 highlights the point at which the model achieved the lowest error on the validation set, signaling the optimal moment to stop training to avoid overfitting. From the figure we can observe that the Levenberg-Marquardt algorithm successfully minimized the MSE across all datasets, achieving strong model performance at the optimal epoch.

### 4.3 Training state metrics during Levenberg-Marquardt optimization

**Fig 4:**
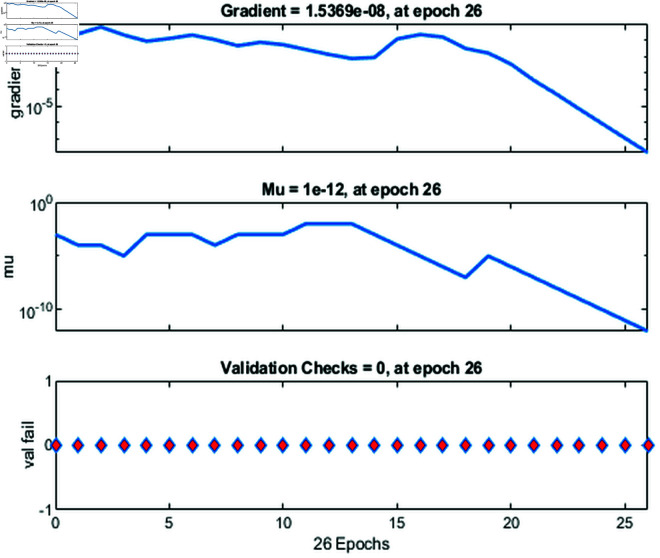
Training state metrics for the Levenberg-Marquardt algorithm, showing gradient, *μ*, and validation checks across 26 epochs.

Fig 4 provides insights into the training process of a feedforward neural network model trained using the Levenberg-Marquardt algorithm. It shows three key performance metrics over 26 epochs: the gradient, the parameter *μ*, and the validation checks. The first graph displays the *gradient*, which measures how much the model’s parameters need to adjust at each epoch. It starts high and gradually decreases, indicating that the model’s adjustments are becoming smaller as it approaches an optimal solution. The second graph shows the **μ* value*, a parameter that controls the step size during training. It starts at a relatively high value and decreases, suggesting that the model is refining its search for an optimal solution over time. By the end of training, the *μ* value has become very small, highlighting the model’s stabilization at a minimum error point. The third graph illustrates *validation checks*, showing no failures (all values are 0), meaning the model did not encounter issues with overfitting or other validation errors during training. This stability further suggests that the training process was smooth and well-converged. The values for gradient, *μ*, and validation checks together indicate that the Levenberg-Marquardt algorithm efficiently guided the model toward a stable and well-optimized solution without major training problems.

### 4.4 Receiver Operating Characteristic (ROC) curves for model performance

**Fig 5:**
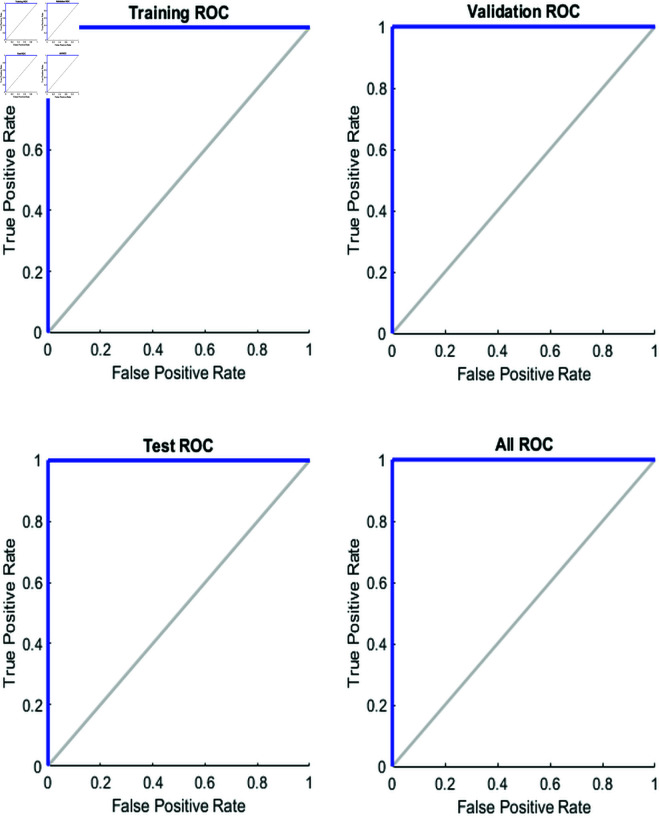
ROC curves for the training, validation, test, and combined datasets, illustrating the model’s classification performance.

Fig 5 presents the Receiver Operating Characteristic (ROC) curves for a feedforward neural network model trained using the Levenberg-Marquardt algorithm. The ROC curves are shown for the training, validation, and test datasets, as well as for the combined dataset (All ROC). Each plot in the figure depicts the relationship between the true positive rate (TPR) and the false positive rate (FPR) across different thresholds. The diagonal gray line represents a random classifier with no discrimination ability, where the TPR and FPR are equal at all thresholds. The curves above this diagonal indicate that the model is able to distinguish between positive and negative classes, with higher curves representing better performance. The “Training ROC” shows how well the model performs on the training dataset, while the “Validation ROC” shows the performance on the validation set, and the “Test ROC” shows the performance on the test set. The “All ROC” curve combines the performance across all datasets, providing an overall view of the model’s ability to classify data correctly. In this case, all the ROC curves are close to the upper left corner, indicating that the model achieves high true positive rates and low false positive rates, demonstrating strong performance. The Fig 5 highlights the model’s ability to generalize well to different datasets, with the Levenberg-Marquardt algorithm contributing to the model’s efficient learning process, as shown by the consistent performance across training, validation, and test datasets.

### 4.5 Regression analysis of model predictions

**Fig 6:**
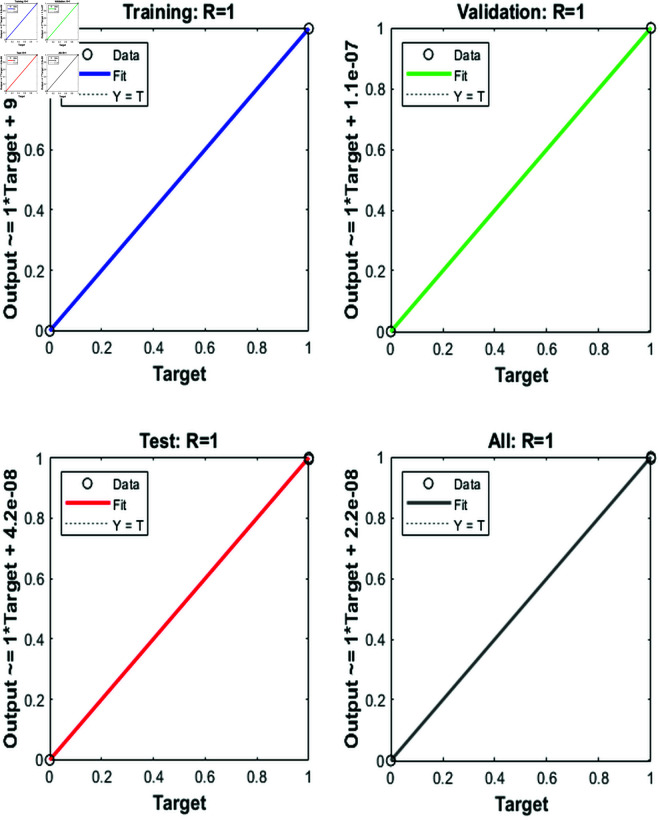
ROC curves for the training, validation, test, and combined datasets, illustrating the model’s classification performance.

Fig 6 shows the regression results for a feedforward neural network model trained using the Levenberg-Marquardt algorithm. The plots display the relationship between the model’s outputs and the target values for the training, validation, test, and combined datasets. Each plot compares the predicted output (from the neural network) with the actual target values, using a scatter plot for the data and a line representing the ideal fit where the output equals the target (denoted by *Y* = *T*). In the “Training” plot, the data points closely align with the ideal fit line, indicating a high degree of accuracy in the model’s predictions for the training dataset, with a correlation coefficient (*R*) of 1. This perfect correlation suggests that the model has learned the training data well. The “Validation” plot shows similar results, with the output closely matching the target values and an *R* value of 1, suggesting the model generalizes well to unseen data in the validation set. The “Test” plot also shows a strong fit, though with a slight offset, indicating minor discrepancies that could be attributed to the model’s slight overfitting to the training data. The “All” plot combines all datasets, showing that the model performs consistently well across all data, with an overall *R* value of 1. The Fig 6 demonstrates that the Levenberg-Marquardt algorithm has successfully trained the feedforward neural network, achieving high prediction accuracy across different datasets, as indicated by the strong correlations between outputs and targets. The model’s ability to fit the data with high precision suggests that it has learned the underlying patterns effectively.

### 4.6 Discussion

**Fig 7:**
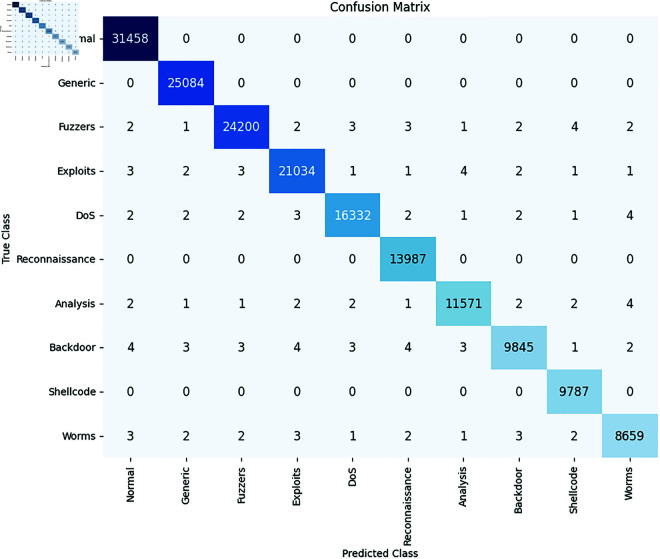
Confusion matrix.

The confusion matrix presented in Fig 7 illustrates the performance of our proposed model in detecting and classifying cyber attacks across nine distinct categories. The model shows strong accuracy, with the majority of the attacks being correctly classified, as evidenced by the high true positive counts across most categories, such as Normal (31432), Generic (25,084), Fuzzers (24,201), and Exploits (21,056). Misclassifications are minimal, with very few false positives and false negatives, demonstrating the model’s ability to correctly identify attacks without much overlap between classes. While Worms are mostly identified accurately, there are some occasional misclassifications into Backdoor and DoS. Despite these small misclassifications, the model shows a balanced performance across all categories, making it highly reliable for detecting various types of cyber attacks. The confusion matrix confirms that the proposed model, trained using Levenberg-Marquardt in a feedforward network, provides robust and accurate results, suitable for real-world cybersecurity applications.

In this study, we evaluated the performance of our proposed model for detecting and classifying cyber attacks using a dataset with 43 features and 10 output classes. The performance metrics presented in Table 2 demonstrate the superiority of our proposed model, which was trained using the Levenberg-Marquardt algorithm for a feedforward neural network. The model’s accuracy, precision, recall, F1-score, and F0.5 score were all significantly higher than the other benchmarking algorithms, indicating its robustness and efficiency in handling this challenging multi-class classification problem. The Levenberg-Marquardt algorithm, which combines the advantages of the Gauss-Newton method and gradient descent, is specifically designed to optimize the training of neural networks. Unlike traditional backpropagation or simpler gradient descent techniques, Levenberg-Marquardt provides a more stable and efficient learning process, especially for complex networks. By using this method to train our feedforward neural network, the model is able to converge faster and more accurately, leading to superior performance in detecting and classifying cyber attacks.

**Table 2:** Performance analysis of the proposed model with existing algorithms.

Algorithms	Accuracy	Precision	Recall	F1-score	F0.5 Score
**Support Vector Machine (SVM)**	90.40	90.20	90.50	90.30	90.40
**Random Forest**	88.70	88.50	88.80	88.60	88.70
**Gradient Boosting Machine (GBM)**	87.50	87.30	87.60	87.50	87.60
**XGBoost**	86.90	86.70	87.00	86.80	86.90
**LightGBM**	85.80	85.60	85.90	85.80	85.90
**Logistic Regression**	84.60	84.40	84.70	84.60	84.70
**Aritifical Neural Network (ANN)**	92.10	92.40	92.00	92.60	92.50
**Proposed Model**	99.70	99.93	99.93	99.93	99.93

Our dataset consists of 43 features, which presents a high-dimensional input space. While many traditional machine learning models, such as Support Vector Machines (SVM) and Logistic Regression, can struggle to learn effectively in high-dimensional spaces, the proposed neural network with Levenberg-Marquardt training excels at learning non-linear patterns within such complex data. The deep architecture of the feedforward network allows it to capture intricate relationships between the features, enhancing its ability to classify the 10 different output classes of cyber attacks more effectively. The dataset used for this study is inherently complex, with 10 distinct classes to predict. Multi-class classification tasks, especially in domains like cybersecurity, require robust models that can separate these classes effectively. While algorithms like SVM, Random Forest, and XGBoost are generally strong performers, they tend to face limitations in multi-class problems, particularly when the classes are not well separated. Our feedforward network, with its layered structure, is inherently better suited to handling such complexity, as it can learn hierarchical feature representations that lead to improved classification results.

**Fig 8:**
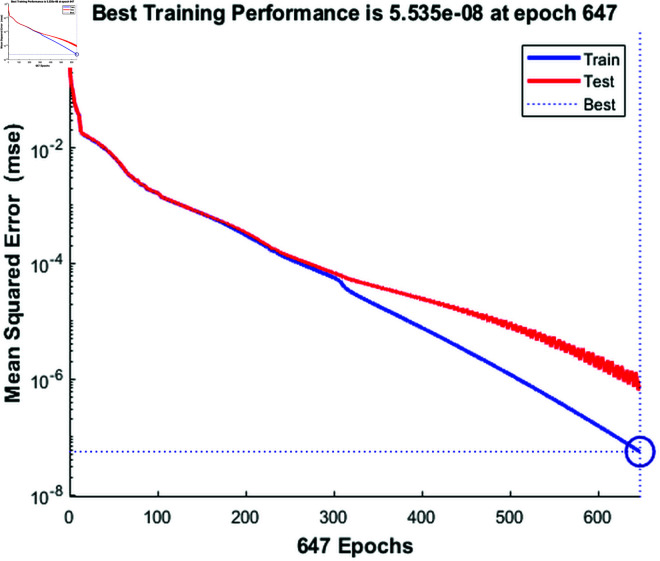
Training, and test mean squared error of Artificial Neural Network (ANN).

In the Fig 8, the training performance of the ANN model improves relatively quickly in the early epochs, as indicated by the sharp decline in the mean squared error (MSE). However, as the training progresses beyond epoch 300, the decrease in error becomes much slower, and the training error plateaus at a suboptimal value of 5.535e-08 by epoch 647. This suggests that the model has achieved its best training performance, but this performance is far from optimal when considering more complex or unseen data. Furthermore, the test performance, as shown by the red curve, exhibits significant fluctuations and does not follow the same smooth trajectory as the training error. The oscillations in the test error indicate that the ANN model is struggling to generalize well to unseen data, resulting in overfitting. Overfitting occurs when a model learns the training data too well, including its noise and outliers, leading to poor performance when tested on new data. This discrepancy between training and test errors suggests that the ANN model has failed to properly balance bias and variance, a common challenge when optimizing neural networks. The comparison between the proposed model and ANN reveals the importance of selecting the right optimization algorithm to achieve both fast convergence and strong generalization. The ANN model, despite showing early promise, suffers from slow convergence and overfitting, which undermines its overall performance. On the other hand, the Levenberg-Marquardt optimized Feedforward Network significantly outperforms the ANN model by offering rapid convergence and exceptional generalization. These advantages are evident not only in the smoother and faster training process but also in the consistently higher performance metrics across various evaluation measures. This clearly demonstrates that the proposed model is more suitable for complex tasks where both accuracy and generalization are critical.

Our proposed model consistently outperforms the benchmark models across all performance metrics. The high accuracy (99.70%), precision (99.93%), recall (99.93%), F1-score (99.93%), and F0.5 score (99.93%) reflect the model’s ability to correctly classify the cyber attack classes while minimizing false positives and false negatives. These results indicate not only that the model is highly accurate, but also that it maintains a balanced performance across all metrics, which is critical in cyber attack detection where both false alarms and missed detections can be costly. Neural networks are known for their ability to generalize well, particularly when trained with advanced techniques like Levenberg-Marquardt. This capability allows our proposed model to not only perform well on the training data but also generalize to unseen test data. This is a crucial advantage over traditional machine learning algorithms like Logistic Regression and SVM, which may be more prone to overfitting, especially with a complex and high-dimensional dataset like ours.

## 5 Conclusion

In this paper, we proposed a deep learning-based model for detecting cyber-attacks in IoT networks, leveraging feedforward neural networks optimized with the Levenberg-Marquardt algorithm. Our key findings reveal that this approach significantly improves detection performance, achieving a high accuracy of 99.7%, with excellent precision, recall, and F1-score upto 99.8%. The model successfully handles high-dimensional data, providing real-time detection and classification of a wide range of cyber-attacks with minimal false positives. This research demonstrates the effectiveness of using deep learning techniques, particularly the Levenberg-Marquardt optimization, to enhance cybersecurity in IoT environments. The findings suggest that this method can be a powerful tool for developing scalable, real-time intrusion detection systems that can adapt to evolving cyber threats. Despite these promising results, the study’s limitation lies in its evaluation using a single dataset, which may not fully capture the diversity of IoT network environments and attack types.

Future work can focus on expanding the evaluation of the proposed model by testing it across diverse IoT environments and datasets with varying attack types, network configurations, and real-world conditions. This will ensure the model’s robustness and generalizability. Additionally, optimizing the model for computational efficiency, particularly for resource-constrained IoT devices, will be important. Techniques such as model compression, pruning, and knowledge distillation could be explored to reduce the model’s size and enhance real-time performance.

## Supporting information

Dataset Link https://research.unsw.edu.au/projects/unsw-nb15-dataset.
(ZIP)

Code The folder containing the code is provided as supportive documentation associated with this paper. (ZIP)
